# Challenges of numerical simulations of cavitation reactors for water treatment – An example of flow simulation inside a cavitating microchannel

**DOI:** 10.1016/j.ultsonch.2021.105663

**Published:** 2021-07-08

**Authors:** Peter Pipp, Marko Hočevar, Matevž Dular

**Affiliations:** Faculty of Mechanical Engineering, University of Ljubljana, Askerceva 6, 1000 Ljubljana, SI, Slovenia

**Keywords:** Cavitation, Computational fluid dynamics, Numerical simulation, Venturi

## Abstract

The research on the potential of cavitation exploitation is currently an extremely interesting topic. To reduce the costs and time of the cavitation reactor optimization, nowadays, experimental optimization is supplemented and even replaced using computational fluid dynamics (CFD). This is a very inviting opportunity for many developers, yet we find that all too often researchers with non-engineering background treat this “new” tool too simplistic, what leads to many misinterpretations and consequent poor engineering.

The present paper serves as an example of how complex the flow features, even in the very simplest geometry, can be, and how much effort needs to be put into details of numerical simulation to set a good starting point for further optimization of cavitation reactors. Finally, it provides guidelines for the researchers, who are not experts in computational fluid dynamics, to obtain reliable and repeatable results of cavitation simulations.

## Highlights

• Researchers with non-engineering background treat CFD too simplistic.
• Poor CFD approach often leads to misinterpretations and poor engineering.
• Cavitation exhibits complex flow features even in the very simplest geometry.
• A detailed example of numerical simulation of cavitation is shown.
• Guidelines for non CFD experts on cavitation modelling are given.

## 1 Introduction

Cavitation i.e., the appearance of vapor cavities inside an initially homogeneous liquid medium, occurs if the pressure is lowered below vapor pressure. The liquid medium is then “broken” at one or several points and “cavities” appear, their shape strongly dependent on the structure of the flow. The vapor structures are unstable, and when they reach a region of increased pressure, they often collapse violently [1]. The research on the potential of cavitation exploitation is currently an extremely interesting topic. Availability of water is becoming an increasing concern in the globalized world, in both developed and developing countries. Therefore, an efficient and clean disinfection technology, such as optimized employment of cavitation, would be readily welcome to substitute or be combined with the existing ones [2].

Different types of cavitation reactors are being promoted by researchers, nowadays. We can generally divide them into i) pump&constriction [3], [4], ii) blow through [5], [6] and iii) rotor–stator type of setups [7], [8], [9]. Most of the advanced lab-scale reactors are the pump&constriction type, where the contaminated water (containing bacteria, virus, algae, etc.) is pushed through the orifice or a Venturi type constriction by the pump, where the sample cavitates. The blow-through setups are essentially the same, but the sample is pushed by compressed air, hence these setups have more controlled conditions, suitable for scientific exploratory studies, but cannot be efficiently used in industrial applications. Finally, the most complex are the rotor–stator devices, which are many times already used in pilot setups.

One of the advantages of hydrodynamic cavitation is its scalability and its potential to be used on an industrial scale. Nonetheless, one must be aware that scaling effects and optimization are not straightforward and inexpensive [10]. Hence, to reduce the costs and time of the optimization process, nowadays, experimental optimization is supplemented and even replaced using computational fluid dynamics (CFD).

The simulation of the physics of cavitating dynamics, which involve simultaneously large density and compressibility variations, turbulence effects, and instabilities at various scales, is still beyond the current state of the art. But in the last 20 years engineers (mostly from the field of turbomachinery) developed reliable methodologies, based on CFD, to predict the essential cavitating flow features [11].

Mastering CFD is a combination of knowledge of fluid mechanics and experience. Especially the latter is a tipping point in the outcome of the study. While understanding the flow inside the flow tract can be relatively easy to master in a reasonable amount of time, the craft of CFD is only learned by a long, hands-on, process. Yet, many researchers, coming from other fields, treat this “new” tool too simplistic, which can lead to many misinterpretations and consequent poor engineering.

Looking at some recent examples of utilizing CFD [12], [13], [14], [15], [16], [17], [18], [19], [20] to interpret the results and facilitate the optimization process reveals that on one side researchers approach the modeling of very complex geometries (rotor–stator interaction, narrow gaps, high-frequency oscillations, swirls), but on the other employ a very rudimentary modeling approach (steady flow approach, very basic turbulence modeling, and even assuming laminar flow conditions and no cavitation modeling). Furthermore, rarely studies of mesh independence and convergence criteria are included in the manuscript, and all too often the boundary conditions are not detailed enough. Since the cavitating flow is an extremely dynamic process, such simulations are incapable of capturing many of its features (for example cloud shedding), which can, later on, be a subject of optimization of the process [2].

In the following sections, we describe the numerical simulation of flow in a very confined geometry – a usually encountered case in cavitation reactors (pump&constriction type). As an example, we have intentionally chosen a very simple reactor, a parallel wall Venturi type microchannel, which is many times used as a starting point in studies of bacteria eradication and virus inactivation by cavitation. On one hand, this very simple and small geometry features predominantly 2D flow. This is a consequence of the laminar flow at the inlet, generally confined geometry, and also the sharp transition from convergent to divergent part of the geometry, which suppresses the effects of secondary flow structures, such as the corner vortices. On the other hand, extremely complex flow conditions appear, and their prediction is essential for further optimization and scale-up.

The aim of the paper is not to directly make advancement in the state of the art in simulations of cavitation, but to i) show the complexity of the flow features that appears even in simple geometry and ii) to explain how the simulation should be performed to capture them. A further goal is to give the researcher, with a background from fields other than (mechanical) engineering, a frame on how to approach simulation of cavitation reactors, how to avoid misinterpretation of the obtained results, and how to report them.

## 2 Venturi reactor and typical flow features

The experimental procedure is only briefly shown here, a much more thorough report can be found in [21].

**Fig. 1:**
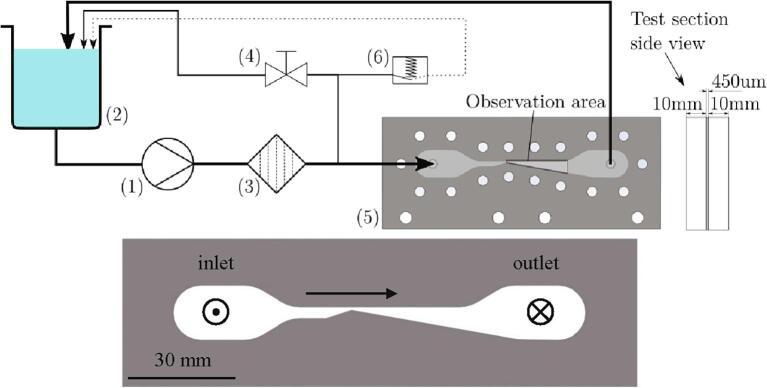
Experimental setup (top) and the Geometry of the Venturi microchannel (bottom).

The experimental set-up is shown in Fig. 1, using planar geometry of the Venturi microchannel, as can be seen from the test section side view in said figure. Microchannels are made of 450 µm thick stainless-steel sheets that include a laser-cut convergent-divergent constriction by sandwiching them between two acrylic glass plates. The convergent angle of the Venturi channel measures 18° and the divergent angle 10°, with the height of the throat being 675 µm. Both entry and exit into and out of the channel are perpendicular to the cross-section plane, with a channel inlet on the left and an outlet on the right side of the channel.

**Fig. 2:**
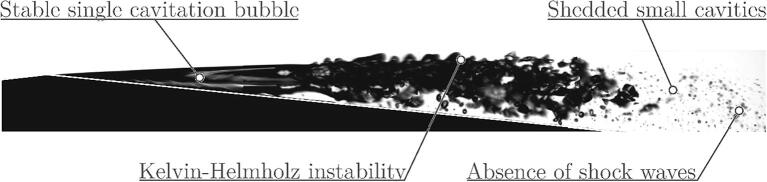
General observations of the phenomena, which are unique for developed cavitation in microchannels [21].

Images of cavitation were captured by high-speed cameras (Photron SA-Z and Photron Mini AX200) at a framerate of typically 200,000 fps in both visible and X-ray light spectrum. Fig. 2 provides a brief overview of the important flow features, which were observed in the microchannel. These are more thoroughly elaborated in a previous paper by Podbevšek et al. [21].

The first appearance of cavitation resembles the condition of supercavitation – a stable large single cavitation bubble, which covers a large portion of the Venturi section. In larger channels, the attached cavity is always composed of numerous cavitation bubbles, while in the smaller channels, the vapor structure consists of a finite number of large (compared to the section size) individual bubbles. What we observe in the present microchannel flow is a large single cavitation bubble stretches from the inception point downstream until the pressure increases well above the saturation pressure. Also, a more detailed observation reveals that its size oscillates periodically and that vaporous structures are shed from its closure.

The interface between the bubble and the liquid jet above becomes “wavy” from time to time, which was identified because of the Kelvin-Helmholtz instability [21]. As the bubble seizes to grow, a shear flow between the bubble interface and the liquid jet above forms. The discontinuity in the velocity induces vorticity at the interface, which becomes unstable, grows, and eventually rolls up into a spiral and causes the separation of numerous small cavitation clouds.

Usually, the frequency of cavitation cloud shedding is associated with the Strouhal number, which for developed cavitating flow typically lies in the range between 0.1 and 0.5 [22]:

(1) $$\mathrm{Str}=\frac{\mathrm{fl}}{v}=0.1\cdots 0.5,$$

where *f* is the cloud shedding frequency, *l* is the size of the cavity and *v* is the reference velocity. In the present case, in a tightly confined geometry, the reversed flow, which normally triggers shedding, cannot fully develop. The cavity, therefore, becomes stable, but on the other hand, the onset of Kelvin-Helmholtz instability still causes cloud separation.

Finally, very seldom a shock wave could be sensed, which is not the case in “normal” size developed cavitating flow, where shocks occur frequently [1]. The reason likely again lies in the very confined geometry, which slows down the collapse of cavities and absorbs the waves.

Even the simplest geometry features a rich ensemble of complex flow features, the most interesting being the Kelvin-Helmholtz instability, which was not reported in the cavitating flow before our study [21]. In the following sections, we describe the setup and results of the simulation, and critically evaluate its capability to reproduce the experimental observation and to give a better look into the physics of flow in the reactor.

## 3 Numerical procedure

CFD software packages offer a wide range of computational models and settings that, along with the increasing computing power of an average computer, enable reliable numerical modeling of engineering problems, including the phenomenon of cavitation. The flow field is described by Navier-Stokes equations, which are a series of partial differential equations that describe how momentum and mass are conserved in a viscous fluid flow. Depending on how we solve these equations and what models we use, we know three main approaches (DNS, LES, and RANS). A direct numerical simulation (DNS), in which Navier-Stokes equations are solved accurately, where the whole range of spatial and temporal scales of the turbulence are resolved. Nonstationary Navier-Stokes equations are solved on an extremely fine computational mesh with correspondingly small time steps to capture even the shortest oscillation periods. Such calculations are thus extremely computationally demanding, requiring a lot of computational resources, which is why this type of method is barely used. The second approach, among them Large-Eddy Simulation (LES) and Detached-Eddy Simulation (DES), have been made possible by increased available computational resources. These relatively new methods are being used in research to accurately resolve larger vortices. The methods involve the spatial filtering of nonstationary Navier-Stokes equations before calculations, which allows larger vortices to pass and rejects smaller ones. The effects on the calculated current (mean current with larger vortices) due to smaller vortices are modeled with a sub-grid scale model. Due to the partial solution of non-stationary equations, there is also a much greater need for computing power. Usually, due to the lack of sufficient computational resources, the third approach, Reynolds-averaged Navier-Stokes (RANS) equations are most used in engineering practice, where we use averaging of arbitrary variables and are thus a simplified form of fluid flow differential equations. In this method, attention is focused on the mean flow and the effects of turbulence on its properties, where turbulent fluctuations are not resolved but are modeled with a turbulence model instead. The results obtained with this kind of approach are accurate enough, given the relatively small required computational resources, that such an approach has been maintained as the main tool for solving engineering problems for the last three decades.

Finally, approaches where the numerical model switches between Eulerian and the Lagrangian mode when the scale is small enough exist [23]. These definitively have potential in the future in various applications, such as cavitation erosion prediction. However, considering the purpose of the present paper – to give an inexperienced cavitation reactor engineer handy guidelines on how to approach CFD modeling of cavitation in their devices, this still lies beyond the scope of this manuscript.

### 3.1 Mesh

**Fig. 3:**
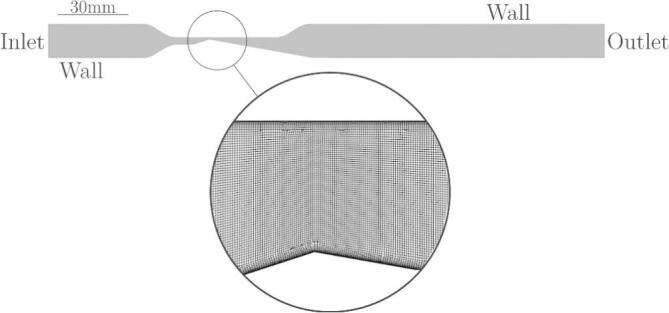
Microchannel computational domain geometry and detail of the mesh in the throat region of the section.

To facilitate the computation of the case and to speed up the calculation of the simulation, we considered the problem two-dimensionally. Thus, the channel entry and exit were extended accordingly, as shown in Fig. 3. The computational mesh was structured. We have tested the mesh independence on 3 meshes. For the mid-course mesh, the discretization error of 0.6% was determined by Richardson extrapolation [24] against the average pressure difference Δ*p* and the length of the cavity *l* at a flow rate of $\dot{m}=$9.03 g/s (Table 1).

**Table 1:** Mesh independence study.

Mesh size	Δ*p* (bar)	*l* (mm)
∼80,000	3.56	25.0
∼160,000	3.71	25.6
∼320,000	3.73	25.7

The final mesh had a constant number of 67 cells along with the channel height. Thus, the height of an individual cell at the site of the throat is 10 µm. The cells increase in proportion to the geometry, with an increase in the length to height ratio towards the inlet and outlet of the domain. Also, we considered the boundary layer, where we determined the total thickness of the boundary layer 50 µm, with 10 layers and a growth rate of 1.2, which is a fine enough grid to keep the *y +* values below 5 in all cases. This means that the first cell along the microchannel wall is located in the viscous sublayer region of a turbulent boundary layer. The computational mesh with which we performed the final numerical simulations counts approx. 160,000 elements (detail in Fig. 3).

### 3.2 Reynolds-averaged Navier-Stokes (RANS) equations

Differential equations, which accurately describe the flow field, require a huge amount of computing power, making them not suitable for practical use. Thus, in practice, we use averaging of variables, namely we define them as the sum of the time-average or mean component Ф and a time-varying fluctuating component *φ'*(*t*) with zero mean value. The written form of the equations is often used in CFD packages. In the equations below, the overline above the variable indicates time-averaged, while the tilde indicates a density-weighted averaged or Favre-averaged variable [25]. The continuity equation is thus:

(2) $$\frac{\partial \overset{-}{\rho }}{\partial t}+\nabla ∙\left(\overset{-}{\rho }\overset{\;}{U}\right)=0,$$

and the momentum equation in the *x* and *y*-axis respectively:

$$\frac{\partial \left(\overset{-}{\rho }\overset{\;}{U}\right)}{\partial t}+\nabla ∙\left(\overset{-}{\rho }\overset{\;}{U}\overset{\;}{U}\right)=$$

(3) $$=-\frac{\partial \overset{-}{P}}{\partial x}+\nabla ∙\left(\mu \nabla \overset{\;}{U}\right)+\left[-\frac{\partial \left(\overset{-}{\overset{-}{\rho }{u^{\text{'}}}^{2}}\right)}{\partial x}-\frac{\partial \left(\overset{-}{\overset{-}{\rho }u^{\text{'}}v^{\text{'}}}\right)}{\partial y}-\frac{\partial \left(\overset{-}{\overset{-}{\rho }u^{\text{'}}w^{\text{'}}}\right)}{\partial z}\right]+S_{\mathrm{Mx}},$$

$$\frac{\partial \left(\overset{-}{\rho }\overset{\;}{V}\right)}{\partial t}+\nabla ∙\left(\overset{-}{\rho }\overset{\;}{V}\overset{\;}{U}\right)=$$

(4) $$=-\frac{\partial \overset{-}{P}}{\partial y}+\nabla ∙\left(\mu \nabla \overset{\;}{V}\right)+\left[-\frac{\partial \left(\overset{-}{\overset{-}{\rho }u^{\text{'}}v^{\text{'}}}\right)}{\partial x}-\frac{\partial \left(\overset{-}{\overset{-}{\rho }{v^{\text{'}}}^{2}}\right)}{\partial y}-\frac{\partial \left(\overset{-}{\overset{-}{\rho }v^{\text{'}}w^{\text{'}}}\right)}{\partial z}\right]+S_{\mathrm{My}}.$$

Some authors stress that for the accurate capturing of details of cavitating flow, performing a compressible flow simulation is essential [26], [27], [28]. For this one must additionally solve the energy conservation equation and introduce equations of state for vapor and liquid. However, as it was shown in numerous studies in the past (for example [29], [30]) sufficient accuracy can also be obtained by assuming the incompressible flow, which significantly reduces the computational load of the simulation, or by using an empirical barotropic state law, where compressible effects are taken into account without solving the energy equation, where more about the model is described under cavitation models.

### 3.3 Turbulence modeling

Turbulence causes the occurrence of eddy flows with a wide range of magnitude and time scales of vortices that interact in a dynamic and complex manner. Given the importance of turbulence in engineering applications, it is understandable that much of the research effort is devoted to the development of numerical methods to capture the significant effects resulting from turbulence.

For engineering purposes in most cases, there is no need to accurately solve turbulent fluctuations and we are often only interested in time-averaged flow properties, most turbulent flows can still be modeled by RANS equations, despite more advanced but computationally demanding approaches (for example Large-eddy simulation (LES) and detached-eddy simulation (DES)) exist.

The result of RANS equations is that we obtain an additional term, the so-called Reynolds stress tensor (RST). The most used RANS turbulent models are divided according to the number of additional transport equations that must be solved together with the RANS flow equations to complete the entire system of equations. Turbulent models are used to express Reynolds stresses by an approximation proposed by Boussinesq in 1877 and based on the assumption that there is an analogy between viscous and Reynolds stresses [31]. Solving the system of equations is thus reduced to determining the turbulent viscosity, which is proportional to the length and time scale of turbulence:

(5) $$\mu_{t}\propto \rho \frac{l_{t}^{2}}{t_{t}}.$$

With the so-called two-equation turbulent models (*k-ε* and *k-ω*), which are also the most used and has become the workhorse of practical engineering flow calculations, we can calculate both, a turbulent length and time scale by solving two additional transport or differential equations alongside algebraic equations to describe Reynolds stresses.

The standard *k-ε* turbulent model [32], is a semi-empirical model based on model transport equations for the turbulence kinetic energy *k* and its dissipation rate *ε*. The assumption for the standard *k-ε* model is fully turbulent flow, and the effects of molecular viscosity are negligible. Transport equations for turbulent kinetic energy and its dissipation rate are as follows:

(6) $$\frac{\partial }{\partial t}\left(\rho k\right)+\nabla ∙\left(\rho kU\right)=\nabla ∙\left(\frac{\mu_{t}}{\sigma_{k}}\nabla k\right)+2\mu_{t}S_{\mathrm{ij}}∙S_{\mathrm{ij}}-\rho \varepsilon$$

(7) $$\frac{\partial }{\partial t}\left(\rho \varepsilon \right)+\nabla ∙\left(\rho \varepsilon U\right)=\nabla ∙\left(\frac{\mu_{t}}{\sigma_{\varepsilon }}\nabla \varepsilon \right)+C_{1\varepsilon }\frac{\varepsilon }{k}2\mu_{t}S_{\mathrm{ij}}∙S_{\mathrm{ij}}-C_{2\varepsilon }\rho \frac{\varepsilon^{2}}{k}$$

In the above equations, the first term represents the rate of change of *k* or *ε*, the second term transport by convection, the third transport by diffusion, and the last two terms represent the rate of production and dissipation of *k* or *ε,* respectively. Turbulent viscosity in the *k-ε* model is defined as:

(8) $$\mu_{t}=\rho C_{\mu }\frac{k^{2}}{\varepsilon }.$$

The model includes five constants, which are determined based on extensive data fitting and correspond to a wide range of turbulent flows [25]. With known strengths and weaknesses of the standard *k-ε* model, modifications (RNG *k-ε* [33] and Realizable *k-ε* [34]) have been established to improve its performance.

The standard *k-ω* turbulent model [35] is an empirical model based on model transport equations for the turbulence kinetic energy *k* and the specific dissipation rate or turbulence frequency *ω*. The standard *k-ω* model contains modifications for low-Reynolds number effects, due to which it is valid in the viscous sub-layer of the boundary layer, compressibility, and shear flow spreading but is sensitive to the values of *k* and *ω* outside the shear layer. Turbulent viscosity in the *k-ω* model is defined as:

(9) $$\mu_{t}=\rho \frac{k}{\omega }.$$

Transport equations for turbulent kinetic energy and specific dissipation rate for turbulent flows at high-Reynolds numbers are:

(10) $$\frac{\partial }{\partial t}\left(\rho k\right)+\nabla ∙\left(\rho kU\right)=\nabla ∙\left[\left(\mu +\frac{\mu_{t}}{\sigma_{k}}\right)\nabla k\right]+P_{k}-\beta^{*}\rho k\omega ,$$

where

(11) $$P_{k}=\left(2\mu_{t}S_{\mathrm{ij}}∙S_{\mathrm{ij}}-\frac{2}{3}\rho k\frac{\partial U_{i}}{\partial x_{j}}\delta_{\mathrm{ij}}\right)$$

and

(12) $$\frac{\partial }{\partial t}\left(\rho \omega \right)+\nabla ∙\left(\rho \omega U\right)=\nabla ∙\left[\left(\mu +\frac{\mu_{t}}{\sigma_{\omega }}\right)\nabla \omega \right]+P_{\omega }-\beta_{1}\rho \omega^{2},$$

where

(13) $$P_{\omega }=\gamma_{1}\left(2\rho S_{\mathrm{ij}}∙S_{\mathrm{ij}}-\frac{2}{3}\rho \omega \frac{\partial U_{i}}{\partial x_{j}}\delta_{\mathrm{ij}}\right).$$

In equations (10), (13), the first term represents the rate of change of *k* or *ω*, the second term transport by convection, the third transport by diffusion, and the last two terms represent the rate of production and dissipation of *k* or *ω,* respectively. This model too includes five constants, determined empirically [25].

The standard *k-ω* turbulent model has its weaknesses, that is why other models, similar to the standard *k-ω* model have been established over time. Two of these are the Baseline (BSL) and the Shear-Stress Transport (SST) *k-ω* turbulent models, developed by Menter [36]. The BSL *k-ω* turbulent model effectively blends the robust and accurate formulation of the *k-ω* model in the near-wall region with the freestream independence of the *k-ε* model in the far-field. To achieve this the standard *k-ω* model and the transformed *k-ε* model are both multiplied by a blending function and both models are added together. The blending function is one in the near-wall region, which activates the standard *k-ω* model and zero away from the surface, which activates the transformed *k-ε* model. In addition to this, the SST *k-ω* turbulent model accounts for the transport effects of the principal turbulent shear stress in the modified formulation of the turbulent viscosity. This makes the SST *k-ω* turbulent model more accurate and reliable for a wider range of flows (e.g. adverse pressure gradient flows), *y +* insensitive, and overall, one of the best two-equation eddy-viscosity turbulent model used today.

However, as it turned out, two-equation turbulent models in the basic form do not give correct results in the case of nonstationary cavitation e.g., cavitation with periodic cavitation cloud shedding. Namely, the model appears to predict excessive turbulent viscosity, determined by equations (8), (9), which prevent the reentrant jet from appearing at the rear of the cavitation cloud, causing it to shed. To improve the turbulent model for more realistic nonstationary cavitation modeling, Reboud et al. [37] proposed a modified turbulent model, where they artificially reduced the turbulent viscosity of the mixture in areas with higher vapor fractions or lower mixture densities. The equations of turbulent viscosity (8) and (9) are thus changed to equations (14), (15) respectively, while the density function is defined by equation (16), where indices *l*, *v*, *m* represents liquid, vapor, and mixture:

(14) $$\mu_{t}=f\left(\rho \right)C_{\mu }\frac{k^{2}}{\varepsilon }$$

(15) $$\mu_{t}=f\left(\rho \right)\frac{k}{\omega }$$

(16) $$f\left(\rho \right)=\rho_{v}+\frac{{\left(\rho_{m}-\rho_{v}\right)}^{n}}{{\left(\rho_{l}-\rho_{v}\right)}^{n-1}}n\gg 1$$

Various values were investigated for the exponent *n*, among which the use of a value of 10 was proposed by Coutier-Delgosha et al. [38]. The described modification of the turbulent model was used and validated by several researchers, both in the Venturi and with the hydrofoil [30], [39], [40], [41], [42]. Recently the use of the turbulence model modification was also experimentally evaluated and finally justified [43], [44].

### 3.4 Two-phase flow modeling

For cavitation modeling, we most commonly use the principle of the homogeneous flow of the mixture, where the two-phase flow is considered as a single-phase flow of the liquid–vapor mixture. This allows us to solve only one equation of motion, as we treat the problem as single-phase, but with variable properties of the mixture. The properties of a mixture of liquid and vapor are thus defined by the proportion of the vapor phase, using the model proposed by Bankoff [45]. The density of the mixture is written:

(17) $$\rho_{m}=\alpha \rho_{v}+\left(1-\alpha \right)\rho_{l},$$

and dynamic viscosity as

(18) $$\mu_{m}=\alpha \mu_{v}+\left(1-\alpha \right)\mu_{l}.$$

However, we must be careful with the latter equation, namely, the written equation is an approximation and does not necessarily apply to every case of two-phase flow. In the model of the homogeneous flow of the mixture, the equations of conservation of mass and momentum are solved by the properties of the mixture, and the equation of conservation of the phase fraction must be solved:

(19) $$\frac{\partial }{\partial t}\left(\rho_{v}\alpha \right)+\nabla ∙\left(\rho_{v}\alpha u_{v}\right)=R_{e}-R_{c}$$

where *α* represents vapor volume fraction, and *R_e_* and *R_c_* mass transfer source terms, which account for the mass transfer between the liquid and vapor phases in cavitation and are thus connected to the growth and collapse of the vapor bubbles. Their formulation differs according to the cavitation model used.

### 3.5 Cavitation models

We know several cavitation models with which we can model the evaporation and condensation of liquid and vapor in the homogeneous flow of the liquid–vapor mixture.

The first is a barotropic model that connects the density of the liquid–vapor mixture with the local static pressure. The model assumes pure liquid (*α* = 0) with density *ρ_l_* when the static pressure in the cell is higher than *p*_vap_ + Δ*p* and pure vapor (*α* = 1) with density *ρ_v_* when the pressure is lower than *p*_vap_ – Δ*p*. Both states are associated with a smooth continuous transition, which leads us to the barotropic law of state and can be written by empirical equation [46]. The barotropic model shows good results, but due to the greater sensitivity of numerical algorithms, the simulations are often unstable with hard-to-achieve convergence. Thus, the application of the model requires a lot of experience to properly adjust the density function as a function of local static pressure [47]. The barotropic model was proposed by Delannoy and Kueny [48] and has been used by other researchers in the past [49], [37], [38], [39].

The second type of cavitation model is the mass or volume fraction transfer model, which is based on the transport equation (19), through which we calculate the volume or mass fraction transfer of the liquid or vapor phase. In the model, we operate with two terms that indicate the source and sink of the vapor phase or describe the process of evaporation and condensation. The terms define the local flow conditions, namely the static pressure and velocity and the properties of the fluid i.e., liquid and vapor density, evaporating pressure, and surface tension. Source terms are derived from the Rayleigh-Plesset equation [50], [51], with the higher-order and viscosity terms being neglected. The most used model of mass or volume fraction transfer, which is also used in commercial software packages for CFD, is the so-called full cavitation model, presented by Singhal et al. [52], which, however, can often be unstable and lead to divergence of the simulation.

In recent years, the most used cavitation models in CFD are bubble dynamics models, first described by Kubota et al. [53], where he used a linear part of the Rayleigh-Plesset equation to describe the development of bubble radius as a function of ambient pressure. Bubble radius and bubble number density, however, determine the proportion of the vapor phase and thus the density of the mixture. Based on the Rayleigh-Plesset equation, other cavitation models were derived based on the pressure and bubble radius dependencies [54]. In all cases, however, it is necessary to know certain variables, such as the bubble number density or bubble initial size, which are very difficult to determine. Cavitation models based on bubble dynamics that are commonly used in commercial software packages for CFD are the Schnerr-Sauer [55] and Zwart-Gerber-Belamri models [56], where we will describe the former in more detail below.

Mass transfer source terms are modeled based on the Rayleigh-Plesset [50], [51] equation describing the growth of a single vapor bubble in a liquid:

(20) $$R_{b}\frac{d^{2}R_{b}}{{dt}^{2}}+\frac{3}{2}{\left(\frac{dR_{b}}{dt}\right)}^{2}=\left(\frac{p_{b}-p}{\rho_{l}}\right)-\frac{4\nu_{l}}{R_{b}}{\dot{R}}_{b}-\frac{2\sigma }{\rho_{l}R_{b}},$$

where *R_b_* denotes bubble radius *p_b_* bubble surface pressure, *ν_l_* liquid kinematic viscosity, and *σ* liquid surface tension coefficient. In some cases, higher-order terms are important [57], but commonly these, along with the effects of surface tension and viscosity, can be neglected. The above equation can be simplified to:

(21) $$\frac{dR_{b}}{dt}=\sqrt{\frac{2}{3}\frac{p_{b}-p}{\rho_{l}}}.$$

The above equation provides a physical approach to introduce the effects of bubble dynamics into the cavitation model. As for the Schnerr-Sauer cavitation model, the *R_e_* and *R_c_* mass transfer source terms are defined as:when $p_{v}\geq p$

(22) $$R_{e}=F_{\mathrm{evap}}\frac{\rho_{v}\rho_{l}}{\rho }\alpha \left(1-\alpha \right)\frac{3}{R_{b}}\sqrt{\frac{2}{3}\frac{\left(p_{v}-p\right)}{\rho_{l}}},$$

when $p_{v}\leq p$

(23) $$R_{c}=F_{\mathrm{cond}}\frac{\rho_{v}\rho_{l}}{\rho }\alpha \left(1-\alpha \right)\frac{3}{R_{b}}\sqrt{\frac{2}{3}\frac{\left(p-p_{v}\right)}{\rho_{l}}},$$

where *F_evap_* and *F_cond_* are the empirical calibration coefficients of evaporation and condensation with the default values of the solver used 1 and 0.2, respectively.

To connect the vapor volume fraction to the number of bubbles per volume of liquid *n_b_* Schnerr-Sauer cavitation model uses:

(24) $$\alpha =\frac{n_{b}\frac{4}{3}\pi R_{b}^{3}}{1+n_{b}\frac{4}{3}\pi R_{b}^{3}},$$

where bubble number density *n_b_* is the only parameter that must be determined in this model.

### 3.6 Boundary conditions

Based on the measurements, cavitation in the microchannel was modeled at different mass flows. At the inlet to the computational domain, we prescribed the corresponding velocities calculated from the mass flows according to the equation below, while at the outlet we prescribed an absolute pressure of 1 bar in all cases:

(25) $$v=\frac{\dot{m}}{\rho A}.$$

The turbulence intensity at the inlet was set to 0% since there is a laminar flow for all cases, and at the outlet, the initial backflow turbulent intensity was set to 5%. Also, we defined that only water enters or exits at the inlet and outlet of the computational domain (i.e., the vapor volume fraction is equal to zero). Boundary conditions for walls were set as stationary walls with a no-slip shear condition and a standard wall roughness model. The modeling did not consider the compressibility of water and water vapor, the parameters of which were taken at a temperature of 20 °C.

### 3.7 Physics and solver settings

Setting proper simulation settings is essential, as are the initial conditions from which to start the simulation, so we first calculated simulations for each mass flow under stationary conditions as single-phase steady-state simulations. Results of which were then taken as the initial conditions for further transient simulations. Numerical simulations were performed using time-dependent Reynolds-averaged Navier-Stokes equations. A homogeneous mixture of water and water vapor was considered and a Schnerr-Sauer cavitation model [55] with an evaporation pressure of 2340 Pa and the bubble number density of 10^11^ were used. For the turbulent model, a modified SST *k-ω* model was used, using the turbulent viscosity correction proposed by Reboud et al. [37] and Coutier-Delgosha et al. [38], described above. The PISO algorithm [58] was used to couple the pressure and velocity. Spatial numerical discretization of solving hyperbolic partial differential equations for everything except pressure and volume fraction, we used the Second-order upwind scheme [59], which gives more accurate results with slightly higher consumption of computer resources compared to the First-order upwind scheme. The PRESTO! interpolation scheme was used to discretize the pressure [31], and for the discretization of the volume fraction First-order upwind scheme [59] was used. For transient solutions, time integration was calculated using the Bounded second-order implicit transient formulation.

The convergence criterion was determined by observing the evolution of different flow parameters (absolute pressure at the inlet and outlet, and velocities at the inlet, outlet, and throat of the microchannel) in the computational domain. The monitored flow parameters were always converged after the sum of the imbalance of the transport equations between iterations over all cells in the computational domain fell below 10^-5^ of the iterative numerical solution of the individual equations in each time step of the simulation. The iteration error of less than 0.02% was estimated. The size of the time step was determined by evaluating its influence against the average pressure difference and the cavity length. No difference in these parameters was found if the time step was shorter than 5 µs, but for the sake of observation of Kelvin-Helmholtz instability a shorter one – 1 µs was eventually used.

For each case, we performed 50 ms of numerical modeling, where the last 30 ms were applicable for further analysis.

## 4 Results

**Fig. 4:**
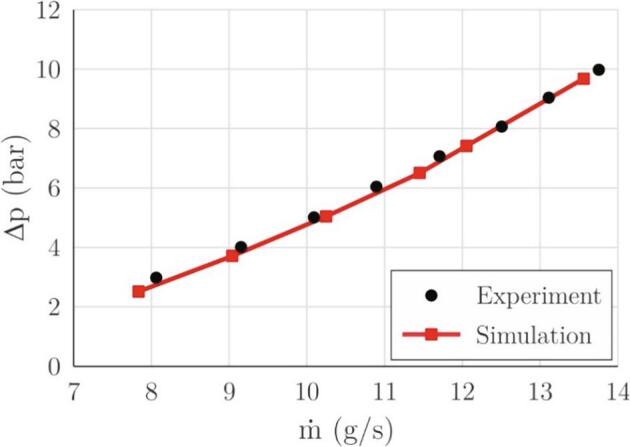
Measured and predicted pressure losses as a function of mass flow rate.

First, the accuracy of simulation in terms of pressure losses is evaluated (Fig. 4) since this is one of the simplest parameters to measure. As the cavitation pocket grows and collapses, the pressure losses in the section oscillate. The values in the diagram (Fig. 4) are an average calculated from 30 ms of flow time (several periods of oscillation).

As shown, the trends fit very well. One can notice a slight underestimation of the pressure losses at small flow rates, which is probably a result of modification of the turbulence model by the artificial decrease of turbulent viscosity parameter (Eq. (15)). It is known that this modification triggers highly unsteady cavitation dynamics prediction even in the cases where it is predominately attached and steady. Nonetheless, based on the comparison of the two curves, one can claim that the simulation predicts the pressure loss in the section adequately.

**Fig. 5:**
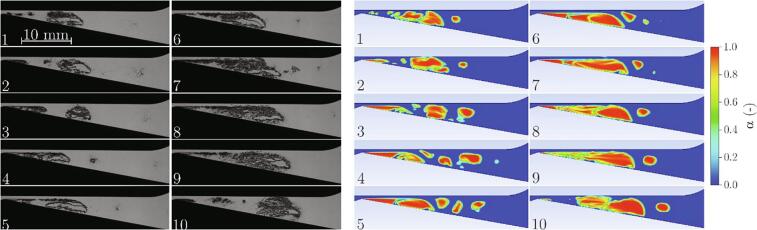
Cavitation cloud shedding (Experiment: $\dot{m}$ = 9.15 g/s, Δp = 4.00 bar, σ = 1.24, Simulation: $\dot{m}$ = 9.03 g/s, Δp = 3.71 bar, σ = 1.26). The time difference between the images is Δt = 0.5 ms.

Cavitation cloud shedding was already mentioned previously. It is one of the most representative phenomena associated with the developed cavitating flow. Simulations are commonly evaluated against their capability of accurately capturing these dynamics. Fig. 5 shows the comparison of the observed and the predicted cavitation cloud shedding process.

Both sequences show one cavitation cloud shedding cycle. They begin at the moment when the attached cavity begins to grow (image 1). At the same time, the previously separated cloud is advected downstream by the flow and collapses (seen in experimental images 5–7). As the attached cavity grows a liquid flow underneath the cavity causes its separation (clearly seen in both experimental and simulation images 8–10). The observed and predicted sizes of the attached cavity and the separated clouds agree well as do times of the distinctive events (growth, separation, collapse) during the process. Simulation on the other hand also predicts the rebound of the collapsed cavitation cloud, which is not seen during the experiment.

As of the topology of cavitation, in experimental images, one can see that, due to the small thickness of the test section, the clouds are almost completely filled by vapor (the transition from water (bright) over the interface (dark) to vapor (bright) is very sharp). This is correctly predicted by the simulation, which shows the very sharp interface and estimates the void fraction *α* inside the cloud close to 1.

**Fig. 6:**
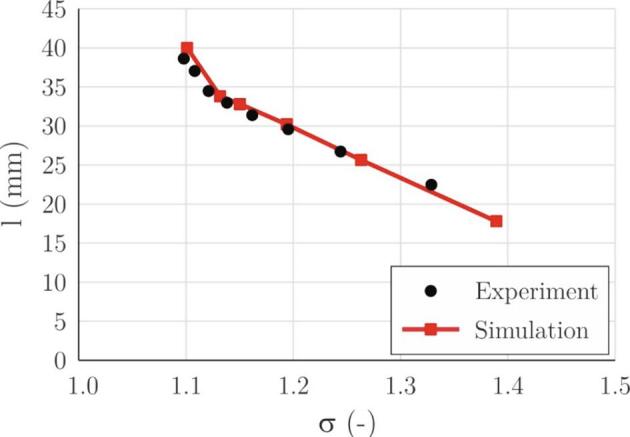
The length of the attached cavity as a function of the cavitation number.

Fig. 6 shows the average cavitation length as a function of the cavitation number. The latter is calculated by [60] as:

(31) $$\sigma =\frac{p_{\mathrm{in}}-p_{v}}{\Delta p},$$

where *p_in_* is the inlet (upstream) absolute pressure, *p_v_* is the vapor pressure and Δ*p* is the pressure difference between the inlet and outlet from the test section.

Again, a good correlation between the experimental and predicted values can be seen. Interestingly the cavity length first increases linearly as the cavitation number is lowered. At *σ* = 1.13 it reaches a bit over 30 mm. This is the position of the curvature of the upper channel wall (see Fig. 5). The change in the pressure gradient causes the change in the size of the cavitation increase, which now begins to grow faster as the cavitation number is decreased. At a cavity length of approximately 40 mm the cavity ceases to grow as choked flow conditions are established.

**Fig. 7:**
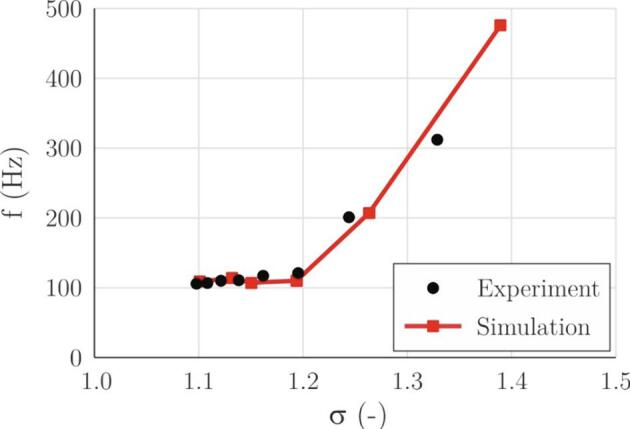
Cavitation cloud shedding frequency as a function of the cavitation number.

**Fig. 8:**
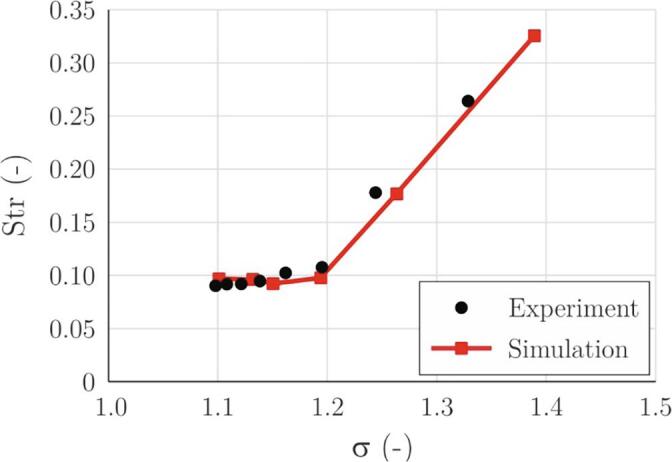
Strouhal number as a function of the cavitation number.

Another important parameter for evaluation of the simulation capability is the cavitation cloud shedding frequency and the corresponding Strouhal number (Fig. 7, Fig. 8). In the experiment, the frequency is evaluated by FFT analysis of the gray level of images inside a region of interest that is positioned at the cavity closure line. In simulations, we do the same for the void fraction. For both cases, the FFT analysis was double-checked by simply counting the void structures.

Looking also at the previous figure (Fig. 6) as the cavitation number decreases, the cavity grows. The flow over it needs a longer time for the passage. It is expected that due to this, the shedding frequency will lower for the larger cavity (smaller cavitation numbers). Interestingly it remains almost constant at approximately 100 Hz below *σ* = 1.2. This is again the effect of a different pressure gradient in the region far away from the throat of the Venturi (>30 mm). In smaller cavity sizes the frequency increases linearly to approximately 500 Hz for the smallest cavity in the present study.

As already mentioned (Eq. (1)) the Strouhal number typically lies in the range between 0.1 and 0.5 [22]. For the present study, it was calculated based on the shedding frequency, the length of the attached cavity, and the velocity at the throat of the Venturi section.

Strouhal number analysis shows the same story as the frequency (Fig. 7). Reassuring is the fact that both experiment and the simulation set their value in the expected range.

**Fig. 9:**
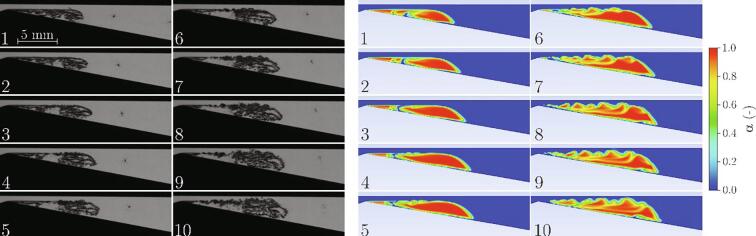
Cavitation cloud separation and the formation of Kelvin-Helmholtz instability (Experiment: $\dot{m}$ = 9.15 g/s, Δp = 4.00 bar, σ = 1.24, Simulation: $\dot{m}$ = 9.03 g/s, Δp = 3.71 bar, σ = 1.26). The time difference between the images is Δt = 40 µs.

We have mentioned in the brief description of the experiment, that onset of the Kelvin-Helmholtz instability was observed. To see whether this phenomenon can be captured by simulation, a more detailed look into the process of cloud separation is shown in Fig. 9. The time difference is 40 μs (an order of magnitude shorter than the one in Fig. 5).

Both the experiment and the simulation show the same story, although the Kelvin-Helmholtz instability is somewhat more pronounced in the case of simulation. The attached cavity grows (1) and reaches its maximal size (2). Shortly later (3) shear flow between the liquid jet and the cavity interface forms. Small changes in the gap between the interface and upper channel wall cause the local changes in the pressure difference on each side of the interface (4), consequently, the gap is locally narrowed or increased further – the waviness increases (5). The shear flow initiates vorticity along with the wavy interface and clear Kelvin-Helmholtz instability forms (6 and 7). Later, the instability engulfs the whole cavitation cloud (8, 9, 10) and causes its dissolvement in the flow (not shown in this sequence). A more detailed analysis with a better insight into the Kelvin-Helmholtz instability and its influence on the cavitation dynamics and cavitation cloud shedding in Venturi microchannels is presented and explained in [21], [61].

## 5 Conclusions

The work presented here was not intended to progress the state of the art in simulations of cavitating flow but to i) serve as an example of how even in a simple geometry very complex fluid dynamics phenomena can occur, ii) how complex an appropriate approach to simulation must be to capture these phenomena and finally iii) how to present and evaluate the simulation results so that they can be further considered a reliable base for optimization of cavitation driven processes.

The present paper serves as an example of how complex the flow features, even in the very simplest geometry, can be, and how much effort needs to be put into details of numerical simulation to set a good starting point for further optimization of cavitation reactors.

Before we conclude we would like to give the following suggestions for future reports, which will (we hope) make the research in the field more transparent and repeatable and will consequently enable faster progress of the science and technology:

1. Effort should be put into an accurate description of the cavitating geometry.
2. If possible, experimental verification should be performed.
3. Computational domain should be meshed with grids of different sizes and a mesh independence test should be performed.
4. Cavitation is a highly unsteady phenomenon and most of its “interesting” features are related to its unsteady nature. Hence it is essential to perform unsteady simulations.
5. Advanced turbulence models or at least appropriate modifications [37], [38] to the existing ones should be used.
6. Governing equations and simulation setup should be clearly described.
7. Results should present both the general behavior of cavitation and integral variables, such as size, shedding frequency, etc.

Hopefully, this work will provide an appropriate foundation for the cavitation reactor engineers, who are not experts in computational fluid dynamics, to set up reliable cavitation simulations that can be further used for reactor optimization.

## CRediT authorship contribution statement

**Peter Pipp:** Investigation, Writing - original draft, Writing - review & editing. **Marko Hočevar:** Supervision, Conceptualization, Writing - review & editing. **Matevž Dular:** Methodology, Conceptualization, Visualization, Writing - review & editing, Supervision.

## Declaration of Competing Interest

The authors declare that they have no known competing financial interests or personal relationships that could have appeared to influence the work reported in this paper.
